# Behavioral factors associated with utilization of healthcare services among elderly in Pakistan: evidence from a nationally representative survey

**DOI:** 10.1186/s12877-021-02005-3

**Published:** 2021-01-12

**Authors:** Lubna Naz, Umesh Ghimire, Abida Zainab

**Affiliations:** 1 Department of Economics, University of Karachi, Karachi, 75270 Pakistan; 2 New ERA, Rudramati Marga, Bagmati, Kalopul, Kathmandu, 44600 Nepal; 3 Poverty Alleviation and Social Safety Division, Centre for Social Entrepreneurship, Islamabad, 46000 Pakistan

**Keywords:** Behavioral factors, Elderly health, Healthcare system, Social protection programs, Wealth quintiles

## Abstract

**Background:**

In Pakistan, health system is facing unprecedented challenges to deal with the healthcare demand of the growing ageing population. Using conceptual framework, this study aims to analyze the factors associated with the utilization of healthcare services in private and public hospitals by the elderly population.

**Methods:**

This study used a sample of 5319 individuals aged 60 and above extracted from the Pakistan Social and Living Standards Measurement Survey 2014–15. We followed the Anderson’s Behavioral model of healthcare utilization. The behavioral factors, including predisposing, enabling and need factors, associated with the use of healthcare care were analyzed using exploratory data analysis and binary logistic regressions. The utilization of healthcare service in the study refers to the visits to private and government hospital.

**Results:**

Out of total 5319 participants around three-fourth or 72.4% of participants visited private hospitals for their healthcare needs. Multivariate analysis showed that older age-group (80 years and above) and participants from urban were 1.35 and 1.53 times more likely to avail healthcare service in private hospitals, respectively. The elderly persons from Khyber Pakhtunkhwa were three times (AOR: 3.29, 95%CI 2.5–4.8) more likely to visit government hospitals than their peers in Punjab. Participants who attended school (AOR: 1.21, 95%CI 0.82–1.31) were more likely to utilize healthcare service in private hospitals. Elders from rich (AOR: 1.04, 95%CI 0.84–1.13) and richest (AOR: 1.29, 95%CI 0.89–1.87) wealth quintiles were more likely to use healthcare in private hospitals. The likelihood of the utilization of healthcare service in private hospitals was 1.7 times higher for three or more consulting visits than the single visit, and 1.5 times higher in the public hospital.

**Conclusions:**

Our findings underscore a dire need for expanding the outreach of healthcare services for the elderly population. It calls for effective implementation of policies which aim at improving equitable access to private healthcare services, and upgrading of government hospitals Moreover, the knowledge generated through this research may be employed to make social protection programs more responsive to age-related healthcare needs, and focused on caregiving for elderly living without spouse.

## Background

The demographic shifts in developing countries, such as increase in life expectancy and decline in fertility rate, have contributed to the growth of elderly populations [1]. The number of people aged 60 years and above is expected to rise by 56% from 901 million in 2015 to 1.4 billion in 2030. The estimated increase in the elderly population is much higher in developing countries than in developed nations [2]. Among others, aging is a single most risk factors that stands out the most in the etiology of disease causation, aggravated by the accumulation of cell damage, compromised immune response and subsequent infirmity to repair cells, and developing a host of different diseases [3, 4]. Several studies have reported that the elderly tend to have several comorbidities [5], chronic health conditions [6], and dependency on multi drugs [7]. These factors contributed to the increased rates of hospital admission and outpatient services utilization by the elders [8].

Taking care of elderly health is a serious undertaking for a responsive health system. The economic impact on elders, responsiveness of health emergencies, and the positive health outcomes are the key components that need to be taken care while dealing with the healthcare of elderly. Private healthcare has emerged as a leading healthcare provider in many low-middle income countries (LMICs) catering healthcare needs of populations [9].

Pakistan is one of the five largest countries in Asia, with a population of 207.7 million. The current population growth rate of 2.4 is higher than all other neighboring countries. By the year 2050, there will be an estimated 26 million people aged 65 years in Pakistan [9]. With an increase in the life expectancy and population growth rate, the percentage of elderly in Pakistan has been growing, resulting in the escalation of dependency ratio in the country. The current dependency ratio of more than 65% is placing a considerable burden on the healthcare system and demand for healthcare and medications [10]. The health care delivery in Pakistan is considered as a neglected sphere of social security. A report indicated that the government allocation in health care is as low as 0.4% of its overall GDP, thereby nearly 78% of the population pays out of their pockets for health services in private health sectors [11].

Pakistan’s healthcare system consists of a mix of public and private healthcare facilities. The services in the public health delivery system range from Basic Health Units (BHU) to tertiary referral centers. The BHU and Rural Health units are the two components of Primary Health Care (PHC) catering health services to the rural population. However, nearly 70% of the population relies on private health facilities, and merely 30% of the population utilize health services in the public healthcare sectors [10]. The community-level health services led by the Lady Health Workers (LHWs), Lady Health Visitors (LHVs), and Community Midwives (CMWs) are recognized as the most significant outreach primary healthcare services in Pakistan [12]. However, these Community-led health services are meant for meeting the reproductive needs of adolescent women in Pakistan.

The use of healthcare services among the elderly depends on a range of factors such as socio-demographic, cultural, financing, the availability of regional resources, etc. [13]. Andersen and J. R Newman proposed a comprehensive framework of the behavioral model for identifying factors of healthcare utilization. This model emphasized that health services utilization is determined by; individual’s predisposition to use services (predisposing factors), factors supporting or hindering service utilization (enabling factors), and patient’s illness level (need factors) [1, 14–16]. The model has been employed widely in the academic research [14, 17, 18]. Some studies have augmented the model by exploring the role of psychosocial factors such as social used norm, knowledge and attitude, and perceived control in determining healthcare utilization [14, 17].

The existing literature identified several factors as the potential barriers for the under-utilization of public healthcare service in Pakistan, including lack of qualified health professionals, poor quality of services, high rates of absenteeism, and inconvenient location of PHC facilities [18]. The responsiveness of public healthcare facilities to elderly healthcare needs remains lower compared to private facilities in Pakistan [18].

Nonetheless, the paucity of studies on the distribution of utilization of public and private healthcare by the elderly indicates a gap in research in Pakistan. There is no clear evidence examining the determinants of the use of outpatient service by the older population in Pakistan. The present study fills the gap by using a modified Anderson’s Behavioral model [16], which is considered a pioneering model to understand healthcare utilization behavior in the developing countries, to highlight the utilization of healthcare services in Pakistan for the older population.

The objective of the present study is to examine the behavioral factors associated with the utilization of healthcare by the elderly and identify the constraints in evaluating an expansive behavioral healthcare utilization model. The utilization of healthcare in our study refers to “realized access to health care” or visits to private healthcare facility or government hospital by the elderly for seeking medical assistance during the illness.

The findings can provide credible evidence to meet the challenges of increasing healthcare needs of an aging population, particularly issues related to socioeconomic and regional disparities in the use of more affordable healthcare services, bifurcation of healthcare as per age of the older persons, and lack of family support for elders living without a spouse/partner in Pakistan and similar settings.

## Methods

### Analytical framework

This paper followed the conceptual framework proposed by Ronald. M. Anderson [16] to analyze the healthcare service access and utilization. According to this model, the population healthcare utilization behavior is determined by three factors: predisposing, enabling, and need. These factors can influence the healthcare utilization at both the individual and contextual level. The predisposing factors include demographic and socioeconomic characteristics of the individual, i.e., gender, age, marital status, place of residence, employment status, social class, attitude, and knowledge of healthcare services. The enabling factors comprise resources that directly or indirectly affect the ability to afford the healthcare services, for example, wealth or income, social security, health insurance etc. The need factors refer to the need to be perceived by ailing person or need to be gauged by a health professional. This study has some data-related limitations; for example, the data is not available on social class, attitudes and knowledge of the elderly, and types of ailment and treatment sought.

**Fig. 1:**
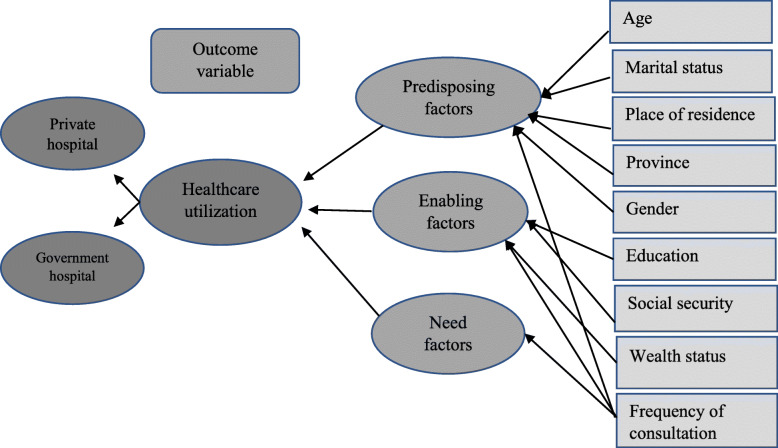
Diagrammatic representation of the analytical framework of healthcare utilization by elderly in Pakistan

Therefore, our behavioral model includes selected predisposing and enabling factors for which the information was available. Further, the dataset did not provide information on the types of ailment, treatment, expenses on medication, rehabilitations, clinical tests, and duration of hospitalization. Therefore, we used the frequency of the consultation as one of the components of the factors of healthcare utilization. The following is a diagrammatic representation of the analytical framework used in the study (Fig. 1). Several previous studies have modified the framework to accommodate the respective contexts of healthcare utilization behavior of the population [14, 16, 19–23].

### Data sources

This study used data from Pakistan Social and Living Standards Measurement (PSLM) Survey (2014–15), the largest administrative dataset sponsored by Pakistan Bureau of Statistics (PBS). The data set is representative at the national, provincial, and district level. The purpose of this survey was to collect information on health, education, assets, and economic condition of households, water, and sanitation, and the satisfaction of households by facilities and services used. PBS has developed its sampling frame for both urban and rural domains segregating each city/town into enumeration blocks, and each block consisted 200 to 250 households with specific boundaries and maps [10].

Given the survey’s objectives, the sample size for the four provinces has been fixed at 5428 sample blocks comprising 81,992 households. This study limits elderly participants from 60 years and above. Given this limitation, a total of 5319 participants were identified and examined for visiting private or government hospital to meet their healthcare needs. The selected sample represents retired personnel from formal sectors as well as working adults aged 60 and above in the informal sector. The data of behavioral factors, such as age, gender, marital status, region, province, education, wealth status, frequency of consultation and pension, of healthcare utilization was considered for empirical analyses.

### Variable description

#### Healthcare services

The study used information on the utilization of healthcare services collected from individuals aged 60 years and above. The information was available on whether the interviewed person fell sick in the past 2 weeks or not, and in case he or she fell sick, did she consult private or government or other healthcare provider for consultation. The “other consultation” included less than 3 % of the overall responses.

The private hospital denotes visits to private dispensary/private hospital, and government hospital implies visits to government hospital/dispensary, rural health unit, basic health unit, and includes others as well. The section D—Health of the PSLM questionnaire and questions 1–3 were used to extract the required data and form a variable. We combined “other consultation” with the government. We used two outcome variables: private hospital visits and government hospital visits. Each outcome variable was measured as a binary response (1/0), where 1 refers to visit(s) s/consultation(s) to/at government hospital/private hospital, and 0 means no visit/no consultation.

#### Behavioral factors

The predisposing factors included gender, region, age groups, province, and marital status. The gender was measured as a categorical variable; male, and female. The place of residence was a categorical variable coded as rural and urban; age groups were categorized in years-group 60–69, 70–79, 80 and above; the province was classified as Punjab, Sindh, Khyber Pakhtunkhwa or KPK, and Balochistan. Marital status comprised two groups, married, and unmarried/widower or widow/ divorced.

The enabling factors were wealth status, pension and attended school. A better wealth status and availability of social security after retirement enhances the ability of the elderly to use healthcare services, and education helps to gain knowledge about the availability and quality of health care services. The different quintiles of the wealth status, a composite index of household asset, was assigned codes in ascending order, poorest, poor, middle, rich, richest. The availability of social security or pension after retirement was categorized as ye and no. Attended school was measured as a binary variable; attended; not-attended.

Moreover, frequency of consultation was used as the component of the factors of utilization of outpatient services, and it was measured by asking the interviewee that how frequently he or she visited the hospital in the last 2 weeks’ prior the survey. The answers were recorded, as follows; only once, two-times, three times, and so on. We grouped all responses into three categories; once; two-times, three times and more.

### Statistical analysis

This study used univariate analysis to examine the background characteristics of the sample. The association between healthcare services and various behavioral factors was analyzed using Chi-square test. Subsequently, a binary logistic regression was used to examine the predicating factors of healthcare services utilization in private or government hospital by the elderly. The model is considered suitable when the dependent/outcome variable, such as the use of healthcare services in private or government hospital, is dichotomized or (0/1). The predicted value was described using adjusted odd ratios (estimated by taking the antilog of the logistic coefficient) at 95% confidence intervals [24]. The results were presented for three significance levels, *P* < 0.05, *P* < 0.010, and *P* < 0.001.

It is pertinent to mention that the logistic model has some advantages over the simple linear probability model. The linear model states that probability is a linear function of the regressors, whereas the binary logistic model asserts that the natural log of the odds is a linear function of regressors. Unlike, the simple linear regression, a binary logistic regression can handle independent variables of any data level (binary, categorical, or ordinal). However, the logistic model is preferred to examine a series of independent variables that can best predict the outcome [25].

## Results

### Univariate analysis

The univariate analyses in Table 1 include the frequency distribution of general characteristics of 5319 study participants disaggregated by the healthcare facility type. Majority of participants (72.4%) visited private hospitals and the remaining visited public hospitals for healthcare services. Out of the total, the highest proportion (53.8%) belonged to the 60–69 age-group, and 54.8% were females followed by subsequent older age-groups and males, respectively. A higher percentage of participants from the rural (83.8), and from Punjab province (44.5%) had realized access to healthcare services in both private and government hospitals, while the utilization of healthcare was rather low among participants from urban and Balochistan.

**Table 1:** Frequency distribution of background characteristics of elderly who make visits to private or government hospital for seeking treatment in 2014–15 in Pakistan (*N* = 5319)

	Private hospital n (%)	Government hospital n (%)	Total n (%)	Chi-square
**Background characteristics**				
**Total**	3853 (72.4)	1466 (27.5)	5, 319	
***Pre-disposing factors***				
**Age group (years)**				0.00
60–69	2059 (71.8)	806 (28.13)	2856 (53.8)	
70–79	1167 (70.73)	483 (29.27)	1650 (31.02)	
80 and above	627 (77.9)	177 (22.01)	804 (15.12)	
**Gender**				
Male	1770 (73.6)	632 (26.3)	2402 (45.16)	
Female	2083 (71.4)	834 (28.5)	2917 (54.8)	
**Place of residence**				0.015
Rural	3181 (71.7)	1251 (28.2)	4432 (83.3)	
Urban	672 (75.7)	215 (24.2)	887 (16.6)	
**Province**				0.000
Punjab	1975 (83.4)	392 (16.5)	2367 (44.5)	
Sindh	671 (65.5)	352 (34.4)	1023 (19.2)	
KPK	785 (59.7)	528 (40.2)	1313 (24.6)	
Balochistan	422 (68.5)	194 (31.4)	616 (11.5)	
**Marital status**				0.72
Married	2048 (72.2)	787 (27.7)	2835 (53.30)	
Widow/divorced/Never married	1805 (72.6)	679 (27.3)	2.484 (46.7)	
***Enabling factors***				
**Attended school**				0.00
Yes	638 (78.19)	178 (21.8)	816 (15.3)	
No	3215 (71.4)	1288 (28.6)	4503 (84.6)	
**Pension**				0.42
Yes	182 (70.2)	77 (29.7)	259 (4.8)	
No	3671 (72.5)	1389 (27.4)	5060 (95.13)	
**Wealth status**				0.003
Poorest	657 (70.9)	269 (29.0)	926 (17.41)	
Poor	716 (70.5)	299 (29.46)	1015 (19.08)	
Middle	729 (70.7)	302 (29.2)	1031 (19.3)	
Rich	785 (70.71)	302 (29.2)	1087 (20.4)	
Richest	966 (76.6)	294 (23.3)	1260 (23.6)	
***Need factors***				0.04
**Frequency of consultation**				
1	1690 (71.9)	659 (28.0)	2349 (44.1)	
2	1569 (72.5)	593 (27.4)	2162 (40.6)	
3 and more	594 (73.5)	214 (26.4)	808 (15.1)	

*P-value* was calculated at three significance levels: **p < 0.010, ***p < 0.001, and *p < 0.05

Note: Private and governmental hospital comprise 100% (row column) and the total category comprises the column percentages

**Fig. 2:**
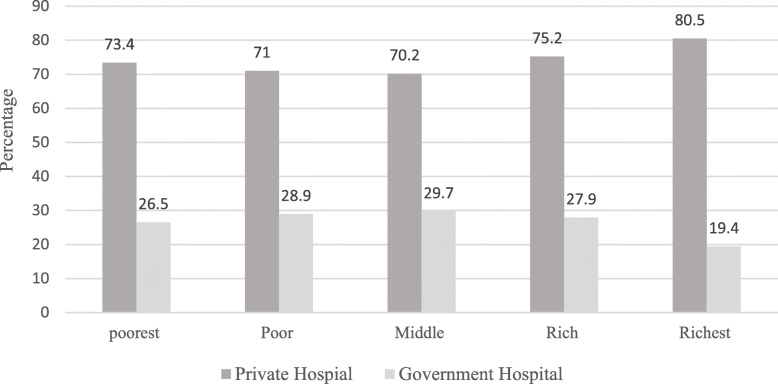
The utilization of healthcare services among elderly associated with their wealth status in Pakistan

**Fig. 3:**
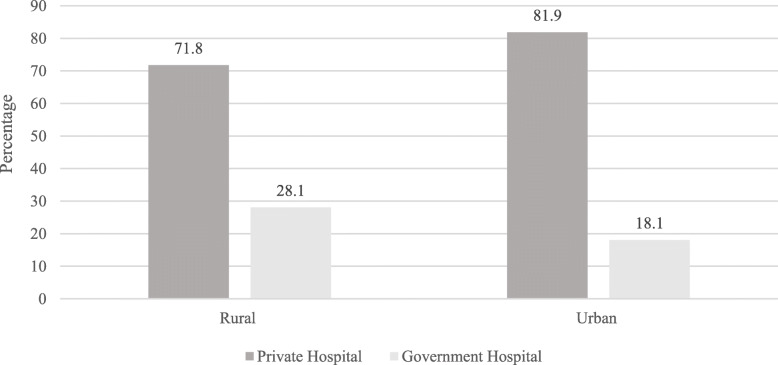
The utilization of healthcare services by elderly in rural and urban areas in Pakistan

Among total participants, 84.6% did not attend school, and 53.3% were married. Overall, 95.1% did not have pension and a wide gap in wealth status among the poorest and richest quintiles was observed among participants. Around 20% (17.4%) of participants were from the poorest wealth quintile whereas 23.6% belonged to the richest wealth quintile. The majority (44.1%) of participants had healthcare service only one time in the reference period, and 40.6% had visited for two times in the last 2 weeks. It is worthwhile to note that, a higher percentage of participants visiting private hospitals were from the wealthier quintiles, whereas, lower percentage of richer participants visited government hospitals. A similar trend was observed in the case of place of residence and healthcare seeking behavior. A higher percentage of participants from the urban visited private hospitals for healthcare services (Figs. 2 and 3).

### Logistic regressions

#### Predisposing factors

The estimates of adjusted odds from Binary Logistic Regressions of the utilization of healthcare services by the elderly are presented in Table 2. Under predisposing factors, participants belonging to older age-group (80 and above vs. 60–69) (Adjusted Odds Ratio (AOR): 1.35, 95%CI 1.38–2.38)), and from urban areas (AOR: 1.53, 95%CI 1.10–2.14) were more likely to visit private hospitals whereas, participants from Sindh (AOR: 1.82, 95%CI 1.4–3.2), KPK (AOR: 3.29, 95%CI 2.5–4.8), and Balochistan (AOR: 1.64, 95%CI 1.3–2.3) were more likely to visit government hospitals for outpatient consultation compared to the participants from Punjab. Gender was not a statistically significant factor of healthcare utilization in both private and public hospitals at the national level. It may be due to a lower utilization of healthcare services among the elderly, irrespective of the gender. Consistent findings were observed in both models reporting participants being older age-group, from urban and from Sindh, and KPK and Balochistan were associated with the utilization of service in private hospitals.

**Table 2:** Results of binary logistic regressions of the utilization of healthcare in private and government hospitals by elderly

Behavioral factors	Healthcare services utilization			
	Private hospital		Government hospital	
	AOR	95% CI	AOR	95% CI
*Pre-disposing factors*				
Gender				
Male	Ref		Ref	
Female	0.96	[0.77–1.96]	1.03	[0.38–1.28]
Age group (years)				
60–69	Ref		Ref	
70–79	1.04	[0.82–1.82]	0.96	[0.76–1.21]
80 and above	1.35*	[1.02–1.78]	0.73**	[0.55–0.87]
Place of residence				
Rural	Ref		Ref	
Urban	1.53*	[1.10–2.14]	0.65*	[0.46–0.93]
Province				
Punjab	Ref			
Sindh	0.54***	[0.40–0.73]	1.82***	[1.4–3.2]
KPK	0.30***	[0.23–0.39]	3.29***	[2.5–4.8]
Balochistan	0.60***	[0.43–0.85]	1.64*	[1.3–2.3]
*Enabling factors*				
Attended school				
No	Ref		Ref	
Yes	1.21	[0.82–1.31]	0.82*	[0.56–1.21]
Wealth status				
Poorest	Ref.		Ref	
Poor	0.93	[0.72–1.22]	1.06	[0.89–1.30]
Middle	0.81	[0.61–1.07]	1.22	[0.93–1.61]
Rich	1.04	[0.84–1.13]	1.02	[0.83–1.24]
Richest	1.29	[0.89–1.87]	1.12	[0.74–1.29]
*Other components*				
Frequency of consultation				
1	Ref		Ref	
2	1.06	[0.79–1.21]	0.98	[0.78–1.25]
3 and more	1.17	[0.97–1.24]	1.05	[0.93–1.11]
Constant				
Observations	3853		1, 466	
Wald (Chi2)	139.7		103.4	
Prob>Chi2	0.000		0.000	
Mean VIF (Max)	1.59		1.31	

*p < 0.05, **p < 0.010, ***p < 0.001; AOR means Adjusted Odd Ratios; Ref. implies reference category

#### Enabling factors

Participants who attended school (AOR: 1.21, 95% CI 0.82–1.31) were more likely to utilize service in private hospitals, while participants who did not attend school were 18% less likely to visit government hospitals. Similarly, those participants belonging to the rich (AOR: 1.04, 95% CI 0.84–1.13) and richest (AOR: 1.29, 95% CI 0.89–1.87) wealth quintiles were more likely to visit private hospitals. Surprisingly, similar figures were observed in the utilization of outpatient services in government hospitals by the participants of rich and richest households. However, the strength of association was higher among the richest quintile participants and utilization of outpatient services in private hospitals.

Three or more consulting visits were associated with a higher probability of healthcare utilization in both private and public hospitals compared to the single visit. The likelihood of utilizing healthcare service in private was 17 times higher than a single visit (AOR 1.17, 95% CI:0.97–1.14), while the likelihood of visiting government hospitals was just 5 times higher (AOR:1.05, 95% CI: 0.93–1.11). However, the results for rich, and frequency of consultation for three or more times were not significant.

## Discussion

The aim of this study was to assess the factors determining the utilization of healthcare services by the elderly population in public and private healthcare facilities in Pakistan. For this, we used Andersen’s conceptual framework of healthcare utilization to categorize study variables into predisposing, enabling, and need factors. Some obvious findings such as age-group of participants, place of residence, and province were significantly associated with the utilization of healthcare services. However, no significant association was observed in the case of gender and healthcare seeking behaviors. Enabling factors like school attendance economic status of participants, and the frequency of consultation, were more associated with treatment-seeking behavior in private hospitals.

Our finding showed that the utilization of healthcare service in private hospital was higher. Though the healthcare services in the government-owned facilities in Pakistan is offered at very minimal cost, several underlying factors such as substandard infrastructures, limited specialized health professionals, long waiting time in the public health facilities stymied the utilization of healthcare services in public health facilities [12].

The healthcare utilization varied across geographic regions in Pakistan. Participants from Punjab province were more likely to seek healthcare services in private hospitals. Similarly, participants from the urban were more likely to visit private hospitals for healthcare services. In other words, participants from urban were less likely to visit government healthcare facilities. Usually, private health facilities are urban-centered and are established for profit motives. The low coverage of private healthcare services in rural areas could be one of the prominent factors for the lower utilization of private healthcare services by rural population. On the other hand, residents from urban areas have easy access to both public and private health services. Inconsistent results were found in earlier studies signifying a varied finding on the health-seeking pattern by rural or urban residents [26–28]. Our study finding is comparable with the study from China that showed the utilization of healthcare services was higher among rural population than urban residents [29].

Older people have been included by the Government of Pakistan in social security, healthcare policies, and plans. Initiated in 2015, the National Health Program of the Prime Minister (PMNHP) is a social health security initiative that is being introduced progressively. In the first step, the focus is on people living below the poverty line of USD 2 per day and treatment coverage is for inpatient care only. There are currently two packages of services available; 1) Secondary care-plus PKR 60,000 / family / year coverage for inpatient care, follow-up and referrals. 2) Priority care kit set of PKR 300,000. The latter provides coverage for high-burden diseases such as diabetes mellitus, heart disease, organ failure, and chemotherapy to every registered family per year. The PMNHP is focused on a creative funding mechanism in which federal and provincial governments work together and take advantage of economies of scale to minimize rates and provide recipients with better services. Until March 10, 2019, the health insurance program or Sehat Saholut Card was operational in 49 districts, and one district is added every month. The program has 6.8 million families participating, which corresponds to approximately 37.4 million people. By the year 2021, the aim is to extend coverage in Pakistan to all districts. The Punjab Health Initiative Management Company has been appointed to conduct the PMNHP. With the financial assistance of the German bank, KfW, Khyber Pakhtunkhwa’s Sehat-Sahulat program was launched in four designated districts. Recently, health insurance’s coverage has been expanded to all KPK persons. However, the program is available to poor families, not to all older persons. The health insurance covers only inpatient services [30].

Healthcare services in many resource-poor settings in low- and middle-income countries face similar constraints. The healthcare delivery system in South Africa where 30% of people choose to pay out of their own pocket to attend private sector facilities even though the public sector primary care is free [31].

Due to the psychological need and low-immune system, more elderly population than other age-groups, utilize health services. A study from the European countries reported that a higher proportion of older age-group tend to have multiple morbidities and tend to visit hospitals more often [32]. Our study showed that older age-group (80 years and above vs. 60–69 years) were significantly more likely to visit private healthcare facility for consultation. Our study findings are consistent with other studies depicting a positive association between healthcare utilization by older populations [33]. As opposed to our finding, a study conducted in Hongkong where the healthcare delivery is much efficient, indicated that poor elders more often visited governmental facilities than private service providers [34]. This provides a clear notion that the availability of an efficient healthcare system is key to the utilization of services in the facility. Our study finding did not show a significant result on the gender-wise differences in the utilization of healthcare services. The treatment-seeking behaviors are widely dependent upon the physical and psychological characteristics of an individual nor it is a gender-specific. Nevertheless, a study conducted in China reported that women tend to use more outpatient services than men due to the physical and psychological needs of women [35].

In Pakistan, a higher proportion of residents in both rural and urban consult private health care providers; private hospitals, clinics, and chemist [36]. A study from Pakistan highlighted that the inaccessibility of public health services and limited operation hours in rural parts of the country are the major factors that explain the underutilization of healthcare services in rural areas. The unavailability of health workers at the health facilities makes it more difficult to receive health services in public health facilities [12, 37]. A study also revealed that traditional health providers - *tabbibs* are more common in rural areas where the presence of both private and public healthcare is sparse [36].

The geographical inequalities in the distribution of healthcare facilities, skilled health manpower, between provinces, districts and rural-urban areas are widespread in the country [38]. The national health policy 2001 of Pakistan envisioned to address urban bias in the health sector by extending public healthcare services in rural areas but the situation has not changed despite a long transition [39]. The allocation of healthcare resources in rural areas ought to be prioritize based on the healthcare needs of elderly to balance the unequal distribution of health service in the rural areas.

Among enabling factors, participants who attended school and from richer households were more likely to visit private hospitals for healthcare services. A higher level of education and better economic status are interrelated to each other and can be taken as a proxy measure of affordability as chances are high that educated individuals get a job that pays well which enables them to pay healthcare costs in private hospitals. A study also reported that not having education was associated with higher utilization of healthcare at PHC [33], while another study revealed that some level of education was associated with utilization of health services in private facilities [40].

Low income has been identified as a major risk for illness and death in older people [5, 6]. Wealth is a significant enabling factor that determines the affordability and utilization of healthcare in good health care providers. At the same time, there is an obvious association between financial empowerment and health and well-being. Several studies from LMICs reported an association between economic status and distribution of healthcare utilization [35, 41].

Our study result showed that richer elders were more likely to visit private hospitals than the poor. This depicts a true picture of inequality in healthcare utilization by rich and poor population in Pakistan. Various studies have reported that factors associated with the utilization of healthcare service are largely dependent upon the various aspects of quality of care including privacy [42, 43], and the readiness of services and long waiting hours [43].

Public perception of lower quality and longer waiting time in public hospitals play an important role in determining factors for the use of type of health care. Despite increasing trust on private healthcare providers in developing countries, a study contested that the quality of care at the private facility was found to be dismal [44]. As stated in the study, heavy reliance on less qualified or unskilled health workers in private facilities, poor people spend a greater proportion of their income on healthcare than the rich [45]. An opposing finding was reported by a study conducted in Ghana showing a positive association between wealth and the use of private facilities [40]. A study conducted in Hong Kong also reported that poor elders were more likely to utilize public facilities and fewer private service providers [34].

We included frequency of consultation as a component of the utilization of healthcare services. Our study reported that higher number of consultations was linked with the utilization of health service in private facilities. Due to the high healthcare costs incurred in private health institutions, some elders requiring higher number consultations could also continue to seek healthcare services in private sectors. Usually, healthcare needs of elderly are too complicated with multiple morbidities that require a frequent visits to health facilities [5]. Hence, social security packages for elders such as subsidies in healthcare packages or health insurances to cover the financial burden of elderly health would be beneficial to ease healthcare services.

The study is not without limitations. Cross-sectional nature of this did not allow us to determine cause and effect relationship between factors associated with the utilization of types of healthcare providers. Prospective studies are thus recommended to understand the actual factors that might influence elderly health. Due to financial constraints, this study did not capture some of the important factors influencing the utilization of health care by elders such as having health insurance, health conditions, work status, this has restricted us to study the comprehensive factors affecting healthcare-seeking behaviors of elderly. Most important of all constraints is the unavailability of data on healthcare needs of the population, especially the data on types of ailment, treatments, expenses on medication and hospitalization, healthcare insurance, duration of the hospitalization, availability of subsidized or free medicine for the poor, and services offered by the government hospital upon hospitalization, and community level care giving services for the older persons.

## Conclusions

Our study concluded that factors such as participants belonging to the older age-group, from rural, having poor economic status, and no schooling were associated with the utilization of healthcare services in government hospital. The findings are suggestive that public healthcare facilities are more attractive to poor, rural, and less educated older persons. There is a need to upgrade the public healthcare system, and regulate the private healthcare sector for charging exorbitant fee for the treatment. It can be done through the establishment of upgraded public health units to serve the elderly population in rural areas. Such establishments can be used as a referral point to the higher-level health facilities and address the inequitable distribution of public facilities. Poor elders are more at risk of having multiple morbidities and are usually unable to pay for the consultation fee for the service in private health facilities. The increased outreach and effective Implementation of social welfare programs, particularly healthcare insurance, can be helpful for those who are excluded from accessing healthcare due to financial constraints. Adequate spending on public healthcare by the government ensures a robust functioning of healthcare delivery in the country. The emerging demand for the health needs of the dependent population is ever increasing in Pakistan. Health equipment and skilled health workers are the backbone of the health system and the allocation of dedicated budget to procure and upgrade health technology could meet the demand of healthcare needs of the country.

## Data Availability

The dataset (PSLM, 2014–15) used in this study is available in the public domain; http://www.pbs.gov.pk/node/1826 and can be used for research purposes with proper acknowledgement.

## Abbreviations

AOR: Adjusted Risk Ratio
BHU: Basic Health Units
CMWs: Community Midwives
CI: Confidence Interval
LHVs: Lady Health Visitors
LHWs: Lady Health Workers
LMICs: Low-middle income countries
PBS: Pakistan Bureau of Statistics
PHC: Primary Health Care
PSLM: Pakistan Social and Living Standards Measurement
